# Age-related hearing loss associated with cognitive impairment in the Polish cohort of the PURE study

**DOI:** 10.3389/fnagi.2025.1540803

**Published:** 2025-03-28

**Authors:** Katarzyna Połtyn-Zaradna, Katarzyna Pazdro-Zastawny, Dorota Szcześniak, Alicja Basiak-Rasała, Maria Wołyniec, Katarzyna Zatońska, Tomasz Zatoński

**Affiliations:** ^1^Division of Population Studies and Prevention of Noncommunicable Diseases, Wrocław Medical University, Wrocław, Poland; ^2^Head and Neck Surgery, Department of Otolaryngology, Wrocław Medical University, Wrocław, Poland; ^3^Department of Psychiatry, Wrocław Medical University, Wrocław, Silesian, Poland

**Keywords:** age-related hearing loss, ARHL, presbycusis, mild cognitive impairment, MCI

## Abstract

**Background:**

Currently, dementia is estimated to be the seventh most common cause of death and one of the leading causes of disability and dependency among older people worldwide. The main aim of this study is to analyze the association of presbycusis and cognitive impairment in the study population. Secondary aim is to identify the risk and prevalence of presbycusis taking into account bio- socio-demographic factors among the residents of Wrocław and surrounding villages.

**Methods:**

Data from 891 participants (559 women and 332 men; mean age: 60.7 years; range: 39–81) in the Polish cohort of the PURE study who met the inclusion criteria for the PURE MIND sub-study and who had a cognitive and functional assessment completed (MoCA test, DSST test, TMT parts A and B, CES-D, SAGE test).

**Results:**

Hearing loss was a significant risk factor for cognitive impairment as assessed using the MoCA, DSST, TMT A and TMT B tests. Mild cognitive impairment (MoCA < 26 score) was present in nearly half of those with hearing loss compared to 26.0 per cent of those without hearing loss (*p* = 0.036). Hearing loss increased the chance of MCI almost 1.5-fold [OR 1.34; CI 0.93–1.93]. Multivariate regression analysis showed that those with hearing loss, hypertension and diabetes scored significantly lower (worse) on the MoCA test (by −0.52 points [95% CI −0.99 to −0.06]; −0.48 points [95% CI −0.96 to −0.01] and −0.69 points [95% CI −1.16 to 0.23], respectively). Excessive body weight and diabetes increased the chance of developing hearing loss by more than 1.5-fold [OR 1.64; CI 1.03–2.68; OR 1.59; CI 1.04–2.41, respectively]. Hearing loss was significantly more common among men (22.3%), irrespective of level of education. The MCI was more common among rural residents (54.8% vs. 34.8%) and in participants with lower levels of education.

**Conclusion:**

Age-related hearing loss is a modifiable risk factor for dementia, emphasizing the importance of routine hearing assessments and timely interventions. Integrating hearing loss management with strategies targeting vascular and metabolic health can help mitigate cognitive decline.

## Introduction

Dementia is currently a huge public health challenge especially considering rapidly aging populations. According to the World Health Organization’s (WHO) projections, the number of people aged 60 and over will double in 2050 to 2.1 billion compared to 1 billion in 2019 (World Health Organization, 2024a). Currently, dementia is estimated to be the seventh most common cause of death and one of the leading causes of disability and dependency among older people worldwide (World Health Organization, 2024b). The WHO projects a dramatic increase in global dementia cases from 55 million in 2019 to 139 million by 2050, with Europe’s cases doubling to 18.6 million (Livingston et al., 2020). Hence, the study of new risk factors and the analysis of mediating or moderating factors between neuropathology and cognitive functioning should be a major scientific and societal focus. One risk factor that needs to be analyzed in detail is a hearing deficit. In both the 2020 and 2024 reports on modifiable risk factors for the development of cognitive impairment and dementia, Livingston et al. (2020, 2024) cite substantial evidence for the importance of this factor in the development of dementia-related neuropathology. Other factors include less education (none or primary school only), hypertension, obesity, depression, diabetes, smoking, social isolation, physical inactivity (Livingston et al., 2024).

Age-related hearing loss (ARHL)–also known as presbycusis especially in view of the aging population—represents a significant public health challenge in the 21st century. According to World Report On Hearing 2021 more than 1.5 billion people (nearly 20% of the global population) live with hearing loss and over 65% of people with any degree of hearing loss are aged above 60 years. The prevalence of hearing loss increases with age, rising from 15.4% among people in their 60 s to 58.2% among people aged over 90. It is estimated that by 2050 the number of people with hearing loss will increase to around 2.5 billion (World Health Organization, 2024c). Modifiable risk factors for hearing loss in adulthood and older age include hypertension, diabetes, obesity, dyslipidaemia and smoking (Han et al., 2018; Lin et al., 2020; Mick et al., 2023; Rolim et al., 2018; World Health Organization, 2024c; Yang et al., 2020). Undiagnosed or untreated ARHL can lead to, among other things, impaired communication capacity, social withdrawal, impaired quality of life, depression, cognitive decline and increased dependency in older people (Ciorba et al., 2012; Hamrah et al., 2024; Lin et al., 2019). The identification of modifiable risk factors for ARHL and its prevalence may provide a basis for interventions to prevent or delay the onset of hearing loss and, consequently, to reduce the prevalence of cognitive impairment among older people. Some potential causal pathways in which ARHL contributes to dementia are being investigated, including depletion of cognitive reserve, social isolation and changes in brain structure. The other possibility is that a common mechanism underlies hearing loss and dementia including microvascular changes and presence of another neuropathologic process affecting the brain and cochlea, subsequently leading to dementia and ARHL.

The main aim of this study is to analyze the association of presbycusis and cognitive impairment in the study population. Secondary aim is to identify the risk and prevalence of presbycusis taking into account bio- socio-demographic factors among the residents of Wrocław and surrounding villages.

## Materials and methods

### Overview

The data used in this study come from the Polish cohort of the international Prospective Urban Rural Epidemiology Study (PURE) established in 2007–2010 as well as the sub-study “Covered cerebral ischemia as an early marker of dementia—retro- and prospective evaluation based on Polish population cohort study” with the acronym PURE-MIND (NCN No. 2015/17/B/NZ7-02963) conducted in 2016–2018 based on the Polish PURE cohort. The PURE study is an international cohort study conducted in 27 countries around the world, including Poland. The aim of the PURE study is to prospectively observe the development of chronic non-communicable diseases (NCDs) and their risk factors (including socio-economic, environmental, behavioral and biological) (Teo et al., 2009). The recruitment process of the Polish cohort at the baseline was described by Zatońska et al. (2016). In contrast, the aim of the PURE MIND (Szcześniak et al., 2021) sub-study was to detect relevant risk factors for dementia and to analyze the severity and significance of hidden cerebral ischaemia using a multi-parametric MR study allowing a detailed qualitative and quantitative *in vivo* assessment of cerebral vascular lesions (Szcześniak et al., 2021).

Data from 891 participants (559 women and 332 man; mean age: 60.7 years; range: 39–81) in the Polish cohort of the PURE study who met the inclusion criteria for the PURE MIND sub-study and who had a cognitive assessment as part of the 2016–2018 study were used for this study. Participants who had contraindications to MRI (including mainly pacemakers and other contraindicated body implants, as well as severe claustrophobia), history of stroke or dementia, history of other neurological brain diseases, presence of significant psychiatric illness, residence in a skilled nursing facility and inability to participate in cognitive assessments (e.g., due to aphasia) were excluded from this analysis. The results presented here are part of a scientific grant of the Wrocław Medical University (SUBK.E260.23.001) called “Presbyacusis and cognitive impairment in the population of residents of Wrocław and surrounding villages.” This study has been reviewed and approved by the bioethics committee of the Wrocław Medical University (consent no.: KB–155/2023).

### Demographic and health-related factor assessment

Socio-demographic and health-related data were collected as a part of the PURE study at the baseline, 3-year, 6-year and/or 9-year follow-ups in the urban and rural population of the Lower Silesian voivodeship in Poland between 2007 and 2018. A comprehensive set of socio-demographic variables were collected and were the basis for a detailed analysis of risk factors of hearing lass and cognitive impairment. All participants were examined according to the global PURE study protocol (Teo et al., 2009) at the baseline and every concomitant follow-up. The data included the prevalence of hypertension, diabetes, coronary heart disease, lipid profile, body mass index (BMI), smoking and alcohol consumption. Self-assessment of the prevalence of hearing loss was performed concurrently with the neuropsychological assessment according to the PURE MIND study protocol in 2016–2018 (Szcześniak et al., 2021). Participants answered to the direct question “Do you have a hearing loss?” Additionally, the use of hearing aid device was also recorded, however in the cohort only 16 participants with hearing loss reported using the device. Therefore, due to small numbers we decided not to analyze the groups with and without hearing aid device separately, but combined them into one category.

### Hypertension criteria

Hypertension was ascertained on the basis of (1) self-reported hypertension previously diagnosed by the physician, (2) self-reported anti-hypertensive medication, and/or (3) an average of two blood pressure measurements 140 mmHg systolic BP and/or 90 mmHg diastolic BP (Szuba et al., 2016). Blood pressure measurements were carried out with an automated oscillometric device (Omron Corporation, Tokyo, Japan). The appropriate cuff size has been selected. Participants were advised to sit and rest for 5 min before consecutive blood pressure measurements. The same methodology was applied at the baseline and at 6 or 9-year follow-up.

### Variables related to glucose and lipid metabolism

Diabetes was ascertained on the basis of fasting plasma glucose level ≥ 126 mg/dl (7.0 mmol/l) and/or if they had a history of diagnosed diabetes and/or have been undergoing treatment ever since. Total cholesterol value was considered abnormal when was ≥ 190 mg/dl (≥ 4.9 mmol/L).

### History of cardiovascular diseases

Coronary heart disease was based on self-reported diagnosis of angina, myocardial infarction, coronary artery bypass graft surgery, or percutaneous coronary angioplasty (each category was not separately identified) (Szcześniak et al., 2021).

### Body mass

The body mass of the participants was measured with the use of Tanita Ironman Body Composition Monitor Model BC-554 with accuracy of 0.1 kg. Body mass index (BMI) was calculated as weight (kg) divided by height (m) squared. Subjects were classified into four BMI categories according to the WHO guidelines as being underweight (BMI < 18.5 kg/m^2^), normal weight (BMI 18.5–24.9 kg/m^2^), overweight (BMI 25.0–29.9 kg/m^2^) and obese (BMI ≥ 30.0 kg/m^2^) (Szcześniak et al., 2021).

### Attitudes toward tobacco and alcohol consumption

History of smoking and alcohol use was self-reported. Participants chose one of three possible responses regarding smoking: “I used tobacco products in the past”; “I currently use tobacco products” or “I have never used tobacco products” and drinking alcohol: “I used alcohol products in the past”; “I currently use alcohol products” or “I have never used alcohol products.”

### Cognitive assessment

The cognitive assessment was conducted in the years 2016–2018. The standardized tools used to assess cognitive function follow the harmonization standards of the Canadian Stroke Network and the National Institute of Neurological Disorders and Stroke, and have been used in accordance with the PURE MIND study protocol (Szcześniak et al., 2021).

The MoCA (The Montreal Cognitive Assessment) scale in Polish adaptation was used to detect cognitive impairment. Although there is a recommendation to use a visually presented version of the MoCA for individuals with hearing aids (Sardone et al., 2021), in our study population, only 16 participants used hearing aids, and none of them required a modified version of the test. Therefore, the traditional, verbally administered MoCA was used for all participants. Before administering the MoCA, we ensured that participants were able to hear the instructions properly to minimize the potential impact of hearing impairment on test performance, in line with recommendations regarding cognitive assessment in individuals with hearing loss (Utoomprurkporn et al., 2020). The published cut-off score (< 26 points) identifies mild cognitive impairment (MCI) or dementia with high sensitivity and specificity (Nasreddine et al., 2005). The MoCA assesses following cognitive domains: attention and concentration, executive functions, memory, language, visuo-constructional skills, conceptual thinking, calculations and orientation. The maximum number of points an examinee can obtain is 30. The score above 26 points indicate cognitive health. In the case of education of less than 12 years one point should be added. The score below 26 points was defined as MCI and between 26 and 30 points as the cognitive norm. In all statistical analyses in this publication, MoCA was used in accordance with the PURE MIND study protocol as a psychometric tool to measure overall cognitive functioning (Nasreddine et al., 2005; Szcześniak et al., 2021).

The DSST (Digit Symbol Substitution Test, Wechsler Adult Intelligence Scale, 3rd Edition) is a 2-min test measuring processing speed, working memory, visuospatial processing, and attention. The test score is equal to the sum of correct symbols drawn in a limited time (Szcześniak et al., 2021; Wechsler, 1997).

The TrailMaking Test (TMT parts A and B) was used to measure cognitive flexibility and central executive functioning. TMT-A version requires combining numbers from 1 to 25, while TMT-B version requires combining numbers and letters alternately. The following variables are taken into consideration while completing the test: time of completion for both versions, number of hints and errors in order of digits. The higher the score, the greater the impairment (Gordon, 1972; Szcześniak et al., 2021).

### Functional assessment

The presence of depressive symptoms was assessed according to the PURE MIND sub-study protocol using The Center for Epidemiological Studies Depression (CES-D) in a Polish adaptation. This scale consists of 20 statements that measure the frequency of depressive symptoms experienced in the past week (Ziarko et al., 2014). Higher scores indicate a greater likelihood of depressive symptoms.

The Standardized Assessment of Global activities in the Elderly (SAGE) is a screening tool to recognize and detect early loss of independence in older adults. Higher scores reveal greater impairment (Marzona, 2011; Szcześniak et al., 2021).

### Statistical analysis

Descriptive statistics were presented using mean, standard deviation, median and quartiles for quantitative variables and counts with percentages for qualitative variables.

Relationship between hearing loss and sociodemographic factors and comorbidities was assessed using Mann–Whitney test or Kruskal–Wallis test for 2 or 3 groups, respectively. When necessary. Qualitative analysis was performed using Chi-squared test. *Post hoc* analysis using Holm adjusted pairwise comparison was used when necessary. Analogous analysis was performed to test for relationship between cognitive functions and hearing loss, sociodemographic factors and comorbidities.

A multivariate analysis of the association between hearing loss, sociodemographic factors, and comorbidities on cognitive function was conducted using both linear and logistic regression models. The choice of method depended on whether cognitive function was assessed as an average questionnaire score or as a binary variable based on an established clinical threshold.

Each regression analysis initially included the full model, which was subsequently reduced by eliminating redundant variables while retaining those that contributed meaningfully to model fit.

Given the relatively low number of patients with cognitive impairment, certain variables, such as CHD and lipid levels, were not included. In turn, smoking status was analyzed as a joint category “ever smokers” (including current and former smokers), whereas overweight as joint category “excessive body mass” (including overweight and obesity according to BMI).

All analyses were performed in R for Windows, version 4.4.1 (R Studio Team, 2024). *P* < 0.05 was selected as the significance threshold.

## Results

Characteristics of the study population (*n* = 891) is presented in the Table 1. The majority of participants were women (62.7%), persons between the ages of 49–65 (58.2%), urban residents (77.7%), whit secondary (42.6%) or university education (41.7%). Hearing loss was significantly more common among men (22.3%), irrespective of level of education, and its percentage increased with the age of the subjects (from 3.9% in the < 49 years group to 33.5 in the > 65 years group). The total mean MoCA score was 25.9 ± 2.7 (median 26), and 39.3% of the study participants scored below the recommended cut-off for MCI or dementia (< 26 points). The percentage of MCI or dementia significantly increased with age, was higher among rural residents and decreased with education level. Greater cognitive flexibility, central executive functioning and attention assessed using the DSST test and the TMTA and TMTB test have the youngest tertiary educated individuals. All subjects were characterized as independent after being assessed by SAGE scale (mean score 2.5 ± 2.9, median 2). Loss of independence increases with age and decrease in education level. The average level of depressive symptoms was low (mean score 10.0 ± 8.2, median 8), indicating general lack of depression among the study participants. Higher the average level of depressive symptoms characterized women (mean score 10.9 ± 8.8, median 9) (Table 1).

**TABLE 1:** Characteristic of sociodemographic factors in relation to ARHL, MoCA, DSST, TMT A, TMT B, CES-D and SAGE in the PURE-Poland population.

		Total *N* (%)	Age-related hearing loss			MoCA					DSST		TMT A		TMT B		CES-D		SAGE	
			**Yes**	**No**	***p*-value**	**< 26**	**> 26**	***p*-value**	**Total mean score (SD)**	***p*-value**	**Total mean score (SD)**	***p*-value**	**Total mean score (SD)**	***p*-value**	**Total mean score (SD)**	***p*-value**	**Total mean score (SD)**	***p*-value**	**Total mean score (SD)**	***p*-value**
Total		891	161 (18.1)	730 (81.9)		350 (39.3)	541 (60.7)		25.9 (2.7)		60.5 (14.8)		40.2 (14.7)		90.3 (42.9)		10.0 (8.2)		2.5 (2.9)	
Sex	Female	559 (62.7)	87 (15.6)	472 (84.4)	0.012	218 (39.0)	341 (61.0)	0.8	25.9 (2.7)	0.9	61.9 (14.6)	< 0.001	40.7 (14.4)	0.049	91.9 (42.7)	0.020	10.9 (8.8)	< 0.001	2.6 (3.1)	0.2
	Men	332 (37.3)	74 (22.3)	258 (77.7)		132 (39.8)	200 (60.2)		25.9 (2.7)		58.2 (14.9)		39.3 (15.0)		87.5 (43.1)		8.5 (6.8)		2.2 (2.4)	
Age	< 49	103 (11.6)	4 (3.9)	99 (96.1)	< 0.001	13 (12.6)	90 (87.4)	< 0.001	27.7 (2.1)	< 0.001	75.5 (12.7)	< 0.001	28.9 (7.4)	< 0.001	61.0 (17.4)	< 0.001	9.5 (7.8)	0.5	2.2 (2.8)	0.009
	49–65	519 (58.2)	67 (12.9)	452 (87.1)		199 (38.3)	320 (61.7)		25.9 (2.6)		62.0 (13.6)		38.4 (12.3)		85.7 (34.9)		10.0 (8.4)		2.4 (2.9)	
	> 65	269 (30.2)	90 (33.5)	179 (66.5)		138 (51.3)	131 (48.7)		25.1 (2.7)		51.8 (12.0)		48.0 (16.9)		110.3 (53.5)		10.3 (7.9)		2.7 (2.7)	
Place of residence	Urban	692 (77.7)	127 (18.3)	565 (81.7)	0.7	241 (34.8)	451 (65.2)	< 0.001	26.2 (2.5)	< 0.001	61.9 (15.1)	< 0.001	39.7 (14.1)	0.11	88.7 (42.3)	0.012	10.2 (8.2)	0.2	2.4 (2.8)	0.3
	Rural	199 (22.3)	34 (17.1%)	165 (82.9)		109 (54.8)	90 (45.2)		24.8 (3.0)		55.7 (12.9)		42.0 (16.5)		95.6 (44.6)		9.4 (7.9)		2.7 (3.1)	
Education level	Primary	45 (5.1)	10 (22.2)	35 (77.8)	0.3	33 (73.3)	12 (26.7)	< 0.001	23.3 (3.4)	< 0.001	48.1 (13.9)	< 0.001	50.8 (21.6)	< 0.001	132.2 (63.5)	< 0.001	10.6 (8.7)	0.8	2.8 (3.4)	0.029
	Vocational	94 (10.6)	22 (23.4)	72 (76.6)		63 (67.0)	31 (33.0)		24.3 (2.7)		51.0 (12.3)		45.1 (18.0)		104.4 (50.1)		9.7 (7.3)		3.3 (3.6)	
	Secondary	380 (42.6)	70 (18.4)	310 (81.6)		178 (46.8)	202 (52.2)		25.5 (2.7)		58.7 (13.2)		40.7 (13.7)		94.1 (43.3)		10.3 (8.4)		2.5 (2.9)	
	Higher	372 (41.7)	59 (15.9)	313 (84.1)		76 (20.4)	296 (79.6)		27.0 (2.1)		66.2 (14.5)		37.1 (12.6)		77.6 (30.7)		9.8 (8.1)		2.2 (2.5)	

**FIGURE 1:**
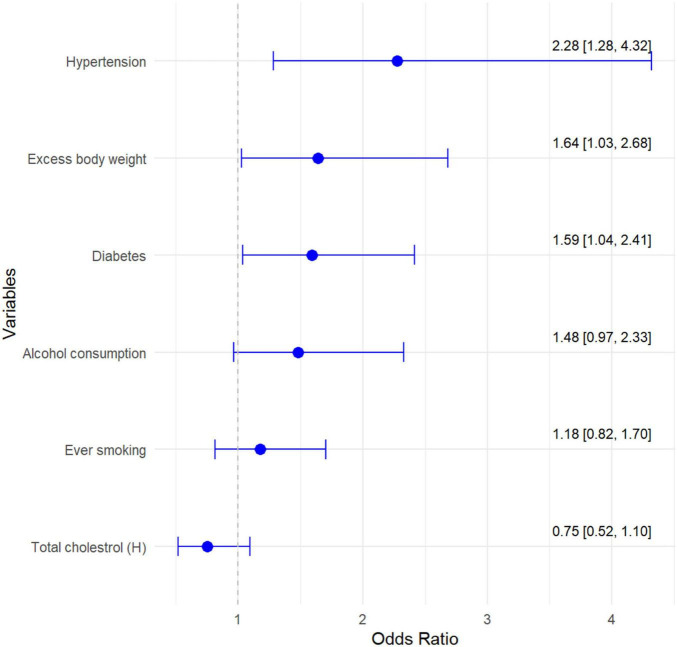
Multivariate model of chance for age-related hearing loss in relation to risk factors.

Among the health-related factors significantly differentiating the prevalence of hearing loss is hypertension. In the hypertension group, hearing loss is present in 20.0% of subjects vs. 8.9% in the group without diagnosed hypertension (Table 2). Hypertension increases the chance of hearing loss by more than 2-fold [OR 2.28; CI 1.28–4.32] (Figure 1). Further factors differentiating the incidence of hearing loss are body weight distribution and diagnosis of diabetes (Table 2 and Figure 1). The percentage of people with hearing loss increases with increasing BMI category. Excess body weight increased and diabetes increased the chance of developing hearing loss by more than 1.5-fold [OR 1.64; CI 1.03–2.68; OR 1.59; CI 1.04–2.41, respectively]. Other health-related factors i.e., smoking, high total cholesterol differentiate statistically significantly the incidence of hearing loss, but were found to be non-significant in multiple regression analysis (Table 2 and Figure 1).

**TABLE 2:** Characteristic of health-related factors in relation to ARHL, MoCA, DSST, TMT A, TMT B, CES-D and SAGE in the PURE-Poland population.

		Niedos łuch			MoCA					DSST		TMT A		TMT B		CES-D		SAGE	
		**Yes**	**No**	***p*-value**	**< 26**	**> 26**	***p*-value**	**Total mean score (SD)**	***p*-value**	**Total mean score (SD)**	***p*-value**	**Total mean score (SD)**	***p*-value**	**Total mean score (SD)**	***p*-value**	**Total mean score (SD)**	***p*-value**	**Total mean score (SD)**	***p*-value**
Age-related hearing loss	Yes	161 (18.1)			75 (46.6)	86 (53.4)	0.036	25.3 (2.6)	0.002	53.3 (12.8)	< 0.001	45.1 (17.2)	< 0.001	104.9 (55.7)	< 0.001	11.1 (8.5)	0.038	3.0 (3.1)	0.002
	No		730 (81.9)		275 (37.7)	455 (62.3)		26.0 (2.7)		62.1 (14.8)		39.1 (13.8)		87.0 (38.8)		9.8 (8.1)		2.4 (2.8)	
Hypertension	Yes	145 (20.0)	564 (80.0)	< 0.001	297 (41.9)	412 (58.1)	0.001	25.7 (2.8)	< 0.001	58.5 (14.2)	< 0.001	41.3 (15.2)	< 0.001	93.1 (43.1)	< 0.001	10.1 (8.2)	0.5	2.6 (2.9)	0.029
	No	16 (8.9%)	164 (91.1)		52 (28.9)	128 (71.1)		26.5 (2.5)		68.5 (14.8)		35.8 (11.2)		79.4 (40.6)		9.8 (8.2)		2.1 (2.8)	
Diabetes	Yes	45 (26.6)	124 (73.4)	0.001	78 (46.1)	91 (53.9)	0.034	25.1 (3.0)	< 0.001	54.6 (13.2)	< 0.001	45.8 (17.2)	< 0.001	104.7 (48.6)	< 0.001	10.9 (9.0)	0.2	3.2 (3.2)	< 0.001
	No	113 (16.0)	595 (84)		264 (37.3)	444 (62.7)		26.1 (2.6)		61.9 (14.9)		38.9 (13.8)		87.1 (41.1)		9.8 (8.0)		2.3 (2.7)	
CHD	Yes	11 (29.7)	26 (70.3)	0.063	15 (40.5)	22 (59.5)	0.9	25.4 (2.3)	0.2	53.9 (14.4)	0.010	43.7 (15.5)	0.2	92.5 (38.0)	0.5	8.7 (6.7)	0.4	2.9 (3.4)	0.6
	No	147 (17.7)	684 (82.3)		328 (39.5)	503 (60.5)		25.9 (2.7)		60.8 (14.7)		40.1 (14.7)		89.8 (43.0)		10.1 (8.2)		2.5 (2.8)	
Total cholesterol	≥ 190 mg/l	94 (16.0)	494 (84.0)	0.023	214 (36.4)	374 (39.5)	0.025	26.0 (2.7)	0.029	62.0 (14.5)	< 0.001	38.7 (13.1)	< 0.001	86.7 (38.5)	0.005	9.9 (8.0)	> 0.9	2.4 (2.9)	0.056
	Normal	64 (22.3)	223 (77.7)		127 (44.2)	150 (55.8)		25.6 (2.8)		57.4 (15.1)		43.4 (17.2)		97.9 (50.0)		10.2 (8.5)		2.6 (2.7)	
Body Mass Index (BMI)	Normal weight	28 (11.0)	226 (89.0)	0.001	76 (29.9)	178 (70.1)	0.001	26.4 (2.5)	< 0.001	65.8 (14.7)	< 0.001	37.3 (13.6)	< 0.001	83.1 (43.0)	< 0.001	10.4 (8.7)	0.8	2.1 (2.5)	0.005
	Overweight	68 (18.8)	293 (81.2)		147 (40.7)	214 (59.3)		25.8 (2.7)		59.5 (14.6)		40.8 (14.6)		90.9 (40.1)		10.1 (8.2)		2.5 (3.1)	
	Obesity	62 (23.4)	203 (76.6)		120 (45.3)	145 (54.7)		25.5 (2.9)		56.8 (13.9)		42.2 (15.5)		96.4 (45.5)		9.6 (7.7)		2.7 (2.7)	
Smoking	Current	18 (13.4)	116 (16.6)	0.007	59 (44.0)	75 (56.0)	0.089	25.7 (2.7)	0.2	58.7 (12.9)	0.005	40.6 (13.8)	0.6	96.7 (48.4)	0.084	11.6 (8.8)	0.014	2.5 (2.9)	0.8
	Former	78 (23.2)	258 (76.8)		195 (58.0)	141 (42.0)		25.8 (2.5)		59.2 (13.8)		39.9 (13.2)		90.2 (36.3)		9.0 (7.4)		2.5 (2.9)	
	Never	65 (15.5)	355 (84.5)		271 (64.5)	149 (34.5)		26.0 (2.8)		62.2 (15.9)		40.2 (16.0)		88.0 (45.3)		10.3 (8.4)		2.4 (2.8)	
Alcohol intake	Yes	124 (19.6)	509 (80.4)	0.072	229 (36.2)	404 (63.8)	0.013	26.0 (2.6)	0.022	61.5 (14.4)	0.008	39.4 (14.1)	0.2	88.5 (41.6)	0.2	10.1 (8.2)	0.2	2.4 (2.8)	0.6
	No	31 (14.2)	124 (19.8)		100 (45.7)	119 (54.3)		25.6 (2.8)		58.6 (15.7)		39.4 (14.1)		91.6 (42.5)		9.5 (8.3)		2.7 (3.2)	

**FIGURE 2:**
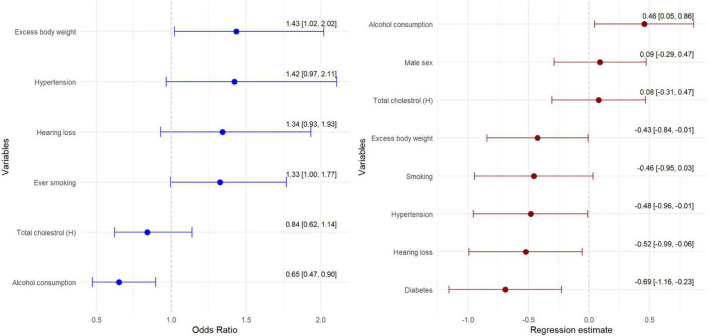
Multivariate model of chance for MCI assessed by MoCA test in relation to risk factors.

**FIGURE 3:**
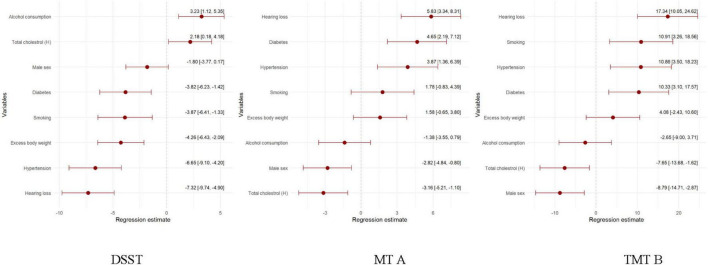
Regression model of the health-related and sociodemographic factors and the occurrence of MCI assessed by DSST, TMT A and TMT B test.

Hearing loss is a significant risk factor for cognitive impairment as assessed using the MoCA, DSST, TMT A and TMT B tests (Figures 2, 3).

**FIGURE 4:**
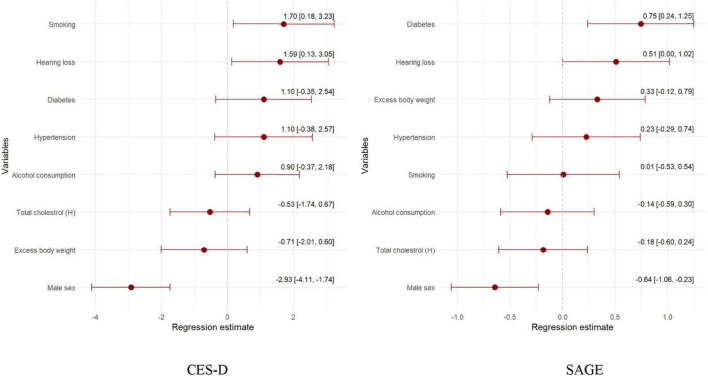
Regression model of the health-related and sociodemographic factors and the occurrence of depression and dependence assessed by CES-D and SAGE.

In addition, it increased the mean depressive symptoms (CES-D) score by 1.57 points and the SAGE score indicating increased dependence by half a point (Figure 4).

Mild cognitive impairment (MoCA < 26 score) is present in nearly half of those with hearing loss compared to 26.0 per cent of those without hearing loss (*p* = 0.036) (Table 1). In addition, hearing loss is a significant factor in lowering the MoCA test score by 0.52 points (Figure 2). Furthermore, a higher prevalence of MCI is found among those with hypertension (41.9% vs. 28.9%), diabetes (46.1% vs. 37.3%), normal total cholesterol (44.2% vs. 36.4%), and not drinking alcohol (45.7% vs. 36.2%). Finally, multivariate regression analysis showed that those with hearing loss, hypertensive and diabetes scored significantly lower (worse) on the MoCA test (Figure 3).

Hearing loss is a factor that significantly reduces cognitive functioning in domains such as processing speed, working memory, visuospatial processing, and attention measured using the DSST and cognitive flexibility and central executive functioning assessed using the TMT A and TMT B test (Tables 1, 2 and Figure 3). In addition to hearing loss, factors reducing processing speed, working memory, visuospatial processing, and attention are excessive body weight, hypertension, diabetes and smoking. Hypertension and diabetes also worsen cognitive flexibility and central executive functioning. In addition, significantly worse TMT B test scores were reported in the ever smoking group (Figure 3).

## Discussion

This study explored the association between age-related hearing loss (ARHL), cognitive impairment, and various sociodemographic and health-related factors among residents of Wrocław and surrounding villages, Poland. The findings underscore the significant public health implications of hearing loss, not only as an isolated condition but also as a modifiable risk factor for cognitive decline. These results are consistent with previous research, highlighting the importance of identifying and addressing risk factors for ARHL to mitigate its impact on cognition and overall quality of life.

Hearing loss, which a common problem in the aging population, may contribute to the risk of decreased cognitive function and subsequently dementia and lower quality of life (Maharani et al., 2018). Decreased hearing may be linked to poorer ability to generate instructional procedures and thus to dementia in a pathomechanism including structural and functioning brain changes, social isolation and poor cognitive load. The dysfunction of cochlea (low fidelity and disturbed encoding of composite sound signals) which is the auditory sensory organ, leads to sensorineural hearing loss including age-related hearing loss or presbycusis, which concerns approximately 40% of persons in the population over 65 years of age (Gates and Mills, 2005). The relationship between hearing loss and cognitive impairments has been a subject of extensive research. Understanding the mechanisms that link these two conditions is crucial for developing effective interventions and improving the quality of life for affected individuals. One of the primary mechanisms proposed is sensory deprivation, where hearing loss leads to reduced auditory input, causing neural degradation and subsequent cognitive decline. This is supported by studies showing that hearing loss can result in decreased synaptic proteins in the hippocampus, making it more vulnerable to damage from factors like amyloid-β, which is associated with Alzheimer’s disease (Chang et al., 2019; Fulton et al., 2015). Additionally, widespread neural degeneration can impact both hearing and cognitive functions, suggesting a common pathological basis (Fulton et al., 2015; Griffiths et al., 2020). Hearing loss increases the cognitive load required for auditory processing, thereby depleting resources available for other cognitive functions. This cognitive resource allocation hypothesis is supported by evidence showing that individuals with hearing loss perform worse on cognitive tasks due to the increased demands on their cognitive resources (Fulton et al., 2015; Griffiths et al., 2020; Uchida et al., 2019). Studies in mice have also demonstrated that moderate hearing loss can lead to declines in spatial working and recognition memory, further supporting this mechanism (Park et al., 2016).

Additionally, hearing loss was associated with higher depressive symptoms and reduced functional independence, as measured by CES-D and SAGE scores, respectively. Hearing loss can lead to social isolation and depression, both of which are significant risk factors for cognitive decline. Depressive symptoms have been shown to mediate the relationship between hearing loss and cognitive impairment, with social relationships moderating this effect. This suggests that improving social interactions and mental health could mitigate some of the cognitive impacts of hearing loss (Cao et al., 2023; Fulton et al., 2015). This finding reinforces the interconnected nature of hearing loss, mental health, and cognitive function, suggesting that interventions targeting ARHL may have wide-ranging benefits for older adults.

The common cause hypothesis suggests that both hearing loss and cognitive decline may stem from a shared underlying pathology, such as age-related neural degeneration. This hypothesis is supported by epidemiological studies that have found a significant association between age-related hearing loss and cognitive decline (Griffiths et al., 2020; Uchida et al., 2019). A study by Jiang et al. (2024) showed that a causal relationship exist between hearing loss, poorer cognitive load and bigger risks of developing different types of dementia. It was revealed that genetically determined sensorineural hearing loss was linked with an elevated risk of Lewy body dementia (*p* = 0.021) and frontotemporal dementia (*p* = 0.035). Genetic predisposition to conductive hearing loss was found to be connected with increased risks of Alzheimer’s disease (*p* = 0.031). Genetically predicted conductive hearing loss and sudden idiopathic hearing loss were causally associated with lower general cognitive performance (*p* = 0.007 and 0.013) and fluid intelligence score (*p* = 0.037 and 0.040). It was also showed that loneliness, depressed mood, and brain cortical volume (especially located in the medial temporal gyrus) may be factors involved in triggering dementia. In our study hearing loss is a significant risk factor for cognitive impairment in the MoCA, DSST, TMT A and TMT B tests. Some studies suggest, however, that hearing loss—particularly in hearing aid users—may be linked to preserved or even enhanced visuospatial abilities, possibly due to compensatory neural mechanisms (Utoomprurkporn et al., 2022). However, findings in this area remain inconsistent, and the extent to which hearing aid use or the duration of hearing loss influences visuospatial performance requires further exploration. In our study, only 16 participants reported using hearing aids, and detailed data on the duration of their hearing loss were not available. Future research should investigate whether hearing aid use or the length of auditory deprivation contributes to variations in visuospatial cognitive performance, as suggested in previous studies (Utoomprurkporn et al., 2022). Understanding these relationships could inform rehabilitation strategies for older adults with hearing loss, particularly in optimizing cognitive and sensory interventions.

The decline in individual’s cognitive abilities is followed by sensory impairment in a mechanism of the reducing the amount of cognitive reserve that can be used to exploit sensory perception (the “perceptual cognitive load” hypothesis), sensory impairment leading to social isolation and thus to sensory decline which results in the subsequent loss of cognitive function (the “sensory deprivation” hypothesis), and both declines are a result of third factors (the “common cause” hypothesis) (Wayne and Johnsrude, 2015). Adults with hearing loss present deficits in their cognitive function (Tun et al., 2012). Liang et al. (2021) in the prospective cohort studies investigating the association between hearing impairment and the incidence of dementia in a community-derived population revealed that hearing loss is likely to be an independent risk factor of dementia, as well as for Alzheimer’s disease in the general adult population. In a longitudinal study in a multiethnic cohort including participants in the Washington Heights-Inwood Columbia Aging Project, revealed that observed hearing loss was associated with 1.69 (95% confidence interval [CI] = 1.3–2.3, *p* < 0.010) times the risk of incident dementia greater risk of incident dementia (Golub et al., 2017). In our study hearing loss was determined as a significant risk factor for cognitive impairment as assessed using the MoCA, DSST, TMT A and TMT B tests.

In our study a higher prevalence of MCI was found among individuals with hypertension (41.9% vs. 28.9%), diabetes (46.1% vs. 37.3%), normal total cholesterol (44.2% vs. 36.4%), and not drinking alcohol (45.7% vs. 36.2%). Hypertension also was increasing the chance of hearing loss by more than 2-fold. Prospective population-based studies (the Copenhagen General Population Study and the Copenhagen City Heart Study) on the Danish general population (111 984 individuals) revealed that there is a connection between high levels of plasma HDL cholesterol and an increased risk of any dementia and its subtypes which was not associated with plasma triglycerides and APOE genotype. The study suggest that to establish independent risk estimation for dementia HDL cholesterol can be used as a plasma biomarker in the earliest targeted prevention strategies (Kjeldsen et al., 2022). Hussain et al. (2024) revealed that very high levels of plasma HDL-C (> 80 mg/dL) are associated with 27% increased risk of dementia in in people > 75 years and older (HR 1.27, 95% CI 1.03, 1.58). The increased dementia risk associated with high HDL-C levels appeared to be independent of traditional dementia risk factors. Considering the fact that circulating HDL-C levels are conveniently measurable and potentially modifiable it may become a dementia biomarker. We revealed that high cholesterol differentiates statistically significantly the incidence of hearing loss.

Studies revealed that higher BMI, and especially obesity, in middle age is a risk factor for the development of dementia (Solas et al., 2017). Kivimäki et al. (2018) evaluated individual-level data from 1,349,857 dementia-free adults from 39 cohort studies assessing Body Mass Index (BMI) at baseline and revealed that in the mean follow-up time 16.1 years (4.3 to 37.7 years range) 6,894 incident dementia cases were recorded. All patients had a higher BMI about 20 years before the first symptoms appeared: each 5-unit increase in BMI was associated with a 16%–33% higher risk of developing dementia. In the period immediately preceding the onset of the disease, the average BMI level of patients was lower compared to the BMI of healthy participants. It was revealed that hazard ratios per 5-kg/m^2^ increase in BMI for dementia were 0.71, 0.94, and 1.16 in a time of BMI assessment: 10 years, 10–20 years, and > 20 years before establishing diagnosis of dementia diagnosis. Higher BMI was connected with increased dementia risk in cases when BMI was evaluated > 20 years before dementia diagnosis. Lower BMI predicted dementia in cases when BMI was estimated < 10 years prior to dementia diagnosis. Two processes (causal and reverse causation) connects BMI and dementia: a direct effect and reverse causation as a consequence of weight loss in the time of the preclinical dementia phase (Kivimäki et al., 2018). The relationship between elevated BMI and increased risk of dementia can also be considered in the context of cardiovascular risk factors. An increase in the number of fat cells in the circulation, along with a decrease in blood supply to the brain, leads to damage to the white matter, which in turn leads to impaired cognitive functions. In addition, some adipokines and inflammatory cytokines in combination with other products of adipose tissue lead to damage to brain tissue (Emmerzaal et al., 2015). Higher BMI may lead to insulin resistance and consequently to type 2 diabetes, which, along with other cardiovascular factors, especially lipid metabolism disorders, is a significant risk factor for dementia. Clarke et al. (2018) showed that, in the course of type 2 diabetes in obese people, similar processes are observed in the hippocampus structures as in the course of Alzheimer’s disease. They also suggest that metabolic defects like dyshomeostasis of glucose metabolism, insulin resistance, and disturbed proteostasis, may be connected with the AD development. A combination of genetic and environmental factors may lead to disturbances in metabolism and consequently increase the risk of AD development by elevating cognitive and non-cognitive symptoms, and neurodegeneration. Our study revealed that the percentage of people with hearing loss increases with increasing BMI category. Excess body weight increases and diabetes increased the chance of developing hearing loss by more than 1.5-fold [OR 1.64; CI 1.03–2.68; OR 1.59; CI 1.04–2.41, respectively]. Dove et al. (2023) evaluated cardiometabolic multimorbidity and incident dementia in a study conducted on 17 913 dementia-free individuals aged ≥ 60 who were observed for 18 years. It was revealed that the association exist between cardiometabolic multimorbidity (especially in mid-life) and an increased risk of dementias.

### Implications for public health interventions

In our study hearing loss was established as a factor that significantly influenced cognitive functioning in domains such as processing speed, working memory, visuospatial processing, and attention. Early diagnosis of hearing loss followed by proper interventions, may lead to decrease number of the burden of functional decline associated with hearing loss. The WHO recommends that starting at age 50, all adults have their hearing screened every 5 years until they are 65 years old. At age 65, they recommend having hearing screening done every 1 to 3 years. The AAO proposes that all adults at age 50 or over should have their hearing tested. The American Diabetic Association introduced similar recommendations about hearing tests for diabetic/prediabetic patients to increase the efficacy of identification. Individuals with hearing loss may benefit form hearing aids in terms of possible slowing down process of cognitive dectine. Cantuaria et al. (2024) state that hearing aids might prevent or delay the onset and progression of dementia. As hearing impairment impacts cognitive function and leads to dementia routine hearing assessment in individuals after midlife should be considered. Introducing a personalized hearing intervention strategies in people with mild cognitive impairment and hearing loss resulted in better outcomes on measures of cognition, mood and quality of life in Yu et al. (2025) study.

The strong association between ARHL and cognitive impairment highlights the need for targeted public health strategies. Screening for hearing loss in high-risk groups, such as older adults with hypertension, diabetes, or obesity, can facilitate early detection and management. Hearing aids and auditory rehabilitation should be central to intervention efforts, alongside lifestyle changes like smoking cessation, weight management, and blood pressure control to reduce the prevalence of both conditions. Promoting social engagement and educational opportunities among older adults may further help preserve cognitive function and quality of life (Nagaraj, 2024).

### Strengths and limitations

This study utilized comprehensive cognitive and auditory assessments in a diverse cohort from both urban and rural settings, supported by validated tools like the MoCA and DSST. However, reliance on self-reported hearing loss may have introduced bias, and the cross-sectional design limits the ability to infer causality. The additional limitation is that our study did not include the assessment of the severity of the hearing loss, nor the duration of the hearing loss or usage of hearing aid device, which can partially influence the visuospatial ability of the participants (Utoomprurkporn et al., 2022). Further longitudinal research is needed to clarify temporal relationships and causal mechanisms.

## Conclusion

Age-related hearing loss (ARHL) is a modifiable risk factor for dementia, emphasizing the importance of routine hearing assessments and timely interventions. Integrating hearing loss management with strategies targeting vascular and metabolic health can help mitigate cognitive decline. Public health policies should prioritize hearing loss screening and interventions as part of dementia prevention efforts. Further longitudinal studies are essential to refine and optimize these strategies.

## Data Availability

The raw data supporting the conclusions of this article will be made available by the authors, without undue reservation.
